# Animal sheltering: A scoping literature review grounded in institutional ethnography

**DOI:** 10.1017/awf.2022.4

**Published:** 2023-01-26

**Authors:** Katherine E Koralesky, Janet M Rankin, David Fraser

**Affiliations:** 1 Animal Welfare Program, University of British Columbia, 2357 Main Mall, Vancouver, BC V6T1Z4, Canada; 2 Faculty of Nursing, University of Calgary, 2500 University Drive NW Calgary, AB T2N1N4, Canada

**Keywords:** animal welfare, compassion fatigue, euthanasia, length of stay, relinquishment, staff

## Abstract

A diverse research literature now exists on the animals, staff and organisations involved in animal sheltering. We reviewed this research through the lens of institutional ethnography, a method of inquiry that focuses on the actual work that people do within institutions. The main topics, identified through a larger ethnographic study of animal sheltering, were: (i) research about shelter staff and officers; (ii) the relinquishment of animals to shelters; and (iii) animals’ length of stay in shelters. After reviewing the literature, we held focus groups with shelter personnel to explore how their work experiences are or are not represented in the research. The review showed that stress caused by performing euthanasia has attracted much research, but the decision-making that leads to euthanasia, which may involve multiple staff and potential conflict, has received little attention. Research on ‘compassion fatigue’ has also tended to focus on euthanasia but a granular description about the practical and emotional work that personnel undertake that generates such fatigue is missing. Published research on both relinquishment and length of stay is dominated by metrics (questionnaires) and often relies upon shelter records, despite their limitations. Less research has examined the actual work processes involved in managing relinquishment as well as monitoring and reducing animals’ length of stay. Institutional ethnography’s focus on people’s work activities can provide a different and more nuanced understanding of what is happening in animal sheltering and how it might better serve the needs of the animals and staff.

## Introduction

For many decades the sheltering and protection of companion animals have been a primary focus of the animal welfare movement. These topics have also stimulated a large body of research that investigates the animals (e.g. Arhant *et al.* 2015; Protopopova 2016), the people (Arluke 1991; Baran *et al.* 2009; Schabram & Maitlis 2017) and the organisations involved (Irvine 2003; Weiss *et al.* 2013). The extant research, however, has tended to concentrate on certain aspects of animal sheltering and protection such as relinquishment of animals by owners and the stress experienced by shelter staff who perform euthanasia. For the most part, the literature does not provide an integrated understanding of how the policies, processes and functioning of the institutions — which govern the everyday work of the staff — determine what happens to animals.

Institutional ethnography (IE) (Smith 1987, 1990, 1999, 2005, 2006) is an approach to inquiry that aims to discover how everyday life and work are shaped and organised within ‘institutions’ which sociologist Dorothy Smith (2005; p 68) defined as “complexes organised around a distinctive function”, with healthcare and education as classic examples. In developing IE, Smith proposed that instead of beginning sociological inquiry by applying existing methods and testing existing theories, inquiry should begin in the actual, concrete experiences of what people actually do. This is what Smith means by discovery. Smith thus called for “a sociology for, not of, people”, and an “ontological shift” (Smith 2005; p 2) toward building knowledge by focusing on the actual, everyday work of people, and explicitly avoiding theorising about what has been observed. IE’s goal is to materially ‘map’ how experiences are organised, especially by institutionalised policies and practices, to happen as they do.

In parallel with its distinctive approach to research, IE researchers use a distinctive approach to reviewing the research literature as a form of discourse analysis, aligned with Michel Foucault’s (1981) interest in language as a tool of social regulation. Smith (1987; p 72–73) identified how the sociological discourse regulates and organises how sociologists conduct research including the language they use and what they try to understand. Within any academic discourse, certain topics, concepts and terms become a focus of attention. Researchers then develop and perpetuate ways of thinking about and interpreting those topics, positioning themselves as observers of a topic, rather than looking at what people are doing.

In this review, we used an IE approach to discourse analysis which identified links between the literature and people’s practice. Smith (1987; p 224) noted that academic discourses not only influence researchers but also are taken up by people doing everyday work practices. Thus, in keeping with IE’s interest in discourse and people’s practice, we also included focus group data from people directly involved in animal sheltering. In the focus groups we presented findings of the review and listened to how well or poorly the research literature captures and reflects the everyday problems people encounter. Thus, in this review, we aimed: (i) to identify and analyse how the academic discourse created by researchers has been shaped; and (ii) to begin to analyse how the knowledge being generated by scholars organises, represents, or glosses over the actual work people are doing in animal welfare organisations.

## Materials and methods

The topics for this review were identified when the primary author (KEK) was collecting data for an IE project in co-operation with the British Columbia Society for the Prevention of Cruelty to Animals (BC SPCA) investigating what happens to companion animals in British Columbia, Canada, when they become involved with the institution of animal sheltering. The literature review covered the breadth of what has been published about shelter staff and officers, together with two other topics — the relinquishment of animals by owners, and the length of time before an animal is adopted. These topics emerged in the workers’ talk and activity as they were observed doing their work and are well-established topics for research.

We structured the review using the theoretical framework of IE with its interest in the social organisation of knowledge, combined with a framework for conducting scoping reviews (Arksey & O’Malley 2005). We also took guidance on how to modify a scoping review for IE from Dalmer (2018, 2020). We searched for peer-reviewed articles in three databases (Ovid Medline, APA PsycInfo and Web of Science) using keywords, subject headings (i.e. phrases used to index articles by concept) and an asterisk to truncate words or phrases. For the first topic (research about staff and officers) the search terms were (people or staff* or employee* or veterinarian* or officer* or volunteer* or worker* OR subject headings ‘Employee Attitudes’ or ‘Employee Characteristics’ or ‘Employee Retention’ or ‘Employee Motivation’ or ‘Veterinary Medicine’) AND (animal shelter* or animal rescu* OR subject headings ‘Housing, Animal’ or ‘Animal Shelters’). For the second topic (animal relinquishment) the search terms were (surrender* or relinquish*) AND (animal shelter* or animal rescu* OR subject headings ‘Housing, Animal’ or ‘Animal Shelters’) AND (pet or pets or ‘companion animal*’ or cat or cats or dog or dogs or rat or rats or rabbit* or bird* or mice or mouse or gerbil* or hamster* OR subject headings ‘Pets’ or ‘animals, domestic or pets’). For the third topic (length of stay in shelters) the search terms were (length of stay OR subject headings ‘Length of Stay’ or ‘Treatment Duration’) AND (animal shelter* or animal rescu* OR subject headings ‘Housing, Animal’ or ‘Animal Shelters’). We did not set date limits, performed searches on November 3, 2020 and set notification alerts for each topic. We also identified references that our searches missed but were cited in other articles and we selected studies for review based on their relevance to the topics.

On the first topic, focused on shelter staff and officers, we excluded articles mostly or solely involving unpaid personnel (e.g. volunteers). We did not include articles about shelter medicine or dog behavioural assessments, as these articles did not focus on the everyday work of people engaged in practicing shelter medicine or conducting assessments. On the topic of relinquishment, we excluded articles on failed adoptions (animals that were returned soon after they were adopted). The final topic ‘length of stay’ included articles that measured ‘live-release rate’ as well as length of stay.

According to Arksey and O’Malley (2005) the aim of a scoping review is to identify prominent themes, patterns and findings on topics. However, an IE-based approach also explores how published research generates an academic discourse whereby new knowledge is explicitly built upon prior research, often using ideas, concepts, theories and methods proposed by earlier researchers. Therefore, in reviewing the literature, we also focused on the foundational ideas, concepts and theories that guide how researchers have approached their investigations, and the methods that have become the accepted ways of doing the research. In this we were following Dalmer (2018), specifically by identifying the established methods that guide the approaches used by researchers (Rankin 2017).

As suggested by Arksey and O’Malley (2005) we implemented a consultation exercise with BC SPCA personnel to gather insights into the problems they face that are not captured in the research literature. We did this through four virtual focus groups in January and February of 2021 (Zoom Video Communications Inc, San Jose, CA, USA 2021). Focus groups lasted 40 to 67 min, and two were with shelter staff (n = 2 and 4), one with animal protection officers (n = 5) and one with senior administrators of the shelters (n = 11). We purposefully included individuals from different work locations in the organisation.

We convened the focus groups to contribute to our discourse analysis whereby, following Smith (2005), we wanted to describe how people in the shelter participated in discourse — that is, whether and how the discourse generated by the research affected how staff think about and interpret their work (Smith 2005; p 224). In each focus group KEK presented the key findings from the literature review and listened for how topics from the literature were taken up by people engaged in sheltering and protecting animals. From the discussion KEK also identified other problems in the everyday work activities that were not covered in the research literature. The University of British Columbia Behavioural Research Ethics Board (#H19-00009-A002) approved the focus groups.

## Results

Following the theoretical approach of IE, we relate each topic of the search to an excerpt of ethnographic data to ground the review in the reality of what people actually do in animal sheltering. We use the excerpts as a tool for examining the concepts and theories discussed in the reviewed literature.

### Shelter staff and officers

*“Euthanasia for behaviour is a grey area. If we get a fearful cat, shelter managers might say, ‘let’s see what happens in a few days’, but I know what that means. I need to get this cat to like people in three days! I would love not to feel like that. I will spend my lunch break with the cat to make it adoptable.” [Focus group comments by a shelter staff member detailing how they make time to help animals that might be euthanased].*

Although we used broad search terms such as ‘people or staff or officers’ AND ‘animal shelter’ to find research on the actual work of shelter staff and officers, most of the research focused on the topic of ‘euthanasia-related stress’, while a small number of articles examined other topics including the presence of women in animal sheltering, animal intake procedures and staff attitudes toward animals.

Euthanasia-related stress is conceptualised in the research literature as a form of stress that arises among shelter staff because of their involvement with euthanasia of animals (for a review, see Scotney *et al.* 2015). This form of stress is widely regarded as a problem in animal sheltering and has become a major topic of research. Despite the early work by Owens *et al.* (1981) who conducted group discussions with euthanasia technicians about their ‘feelings and concerns’ about euthanasia, the interest in euthanasia-related stress has largely been built upon Arnold Arluke’s ethnographic study in an animal shelter in the USA. Arluke conducted observations and interviews and described shelter staff’s experiences, feelings and coping strategies related to performing euthanasia (Arluke 1991; Arluke & Sanders 1996). Arluke coined the term ‘caring-killing paradox’ to describe what he characterised as the clash of feelings that ‘animal people’ (people who love animals and therefore work in animal sheltering) have when institutional practices require them to euthanase animals. The ‘caring-killing paradox’ has become a foundational idea that subsequently influenced a body of research that measures, for example, how staff cope with performing euthanasia (Frommer & Arluke 1999; Baran *et al.* 2009), how performing euthanasia affects the occupational health of staff (White & Shawhan 1996; Rogelberg *et al.* 2007a; Andrukonis & Protopopova 2020), and shelter manager perspectives on euthanasia (Anderson *et al.* 2013).

After Arluke’s study, several other USA-based researchers incorporated the concept of euthanasia-related stress in their research, often using standardised questionnaires to determine how performing euthanasia affects staff depression, burn-out, turnover, or substance use. For example, Reeve *et al.* (2005) measured the extent that animal shelter staff experience euthanasia-related stress through several scales including: (i) the Euthanasia Attitude Scale; (ii) a Work-Family Conflict Scale; (iii) the Hopkins Symptom Checklist (to measure depression); (iv) the Symptom Management Coping Scale (to measure substance use); and (v) a variety of job satisfaction scales. The results showed that staff ‘involved’ with euthanasia had more general job stress, more work-family conflict, greater substance use and overall lower job satisfaction than staff ‘not involved’ with euthanasia. Baran *et al.* (2012) and Lopina *et al.* (2012) surveyed shelter staff using a variety of scales (e.g. Work-Family Conflict Scale, Maslach Burnout Inventory, Brief Cope Scale and Positive and Negative Affect Scale) within the concept of ‘dirty work’ (i.e. occupations or specific work tasks societally viewed as physically, socially or morally dirty or tainted). Baran *et al.* (2012) reported that 40% of staff ‘directly involved’ with euthanasia reported the task to be the most negative part of their job; the majority, however, reported other issues to be the most negative, for example, supervisor-staff conflict and low pay. Lopina *et al.* (2012), interested in whether individual characteristics measured through the scales could predict turnover, had staff complete questionnaires on their first day of work and gathered turnover information two months later. At two months, 28% of staff had voluntarily left their positions and those with more access to job information before starting work (e.g. talking with current staff, visiting the shelter and observing work, asking questions during the interview) were less likely to leave their positions.

Other researchers have used surveys to quantify how shelter staff ‘feel’ about euthanasia. White and Shawhan (1996) surveyed staff and managers on their emotional responses to euthanasia and concluded that individual or group counselling may help alleviate euthanasia-related stress. Rogelberg *et al.* (2007a) reported a positive association between staff turnover and dog euthanasia rates. In addition, Rogelberg *et al.* (2007b) solicited recommendations through an open-ended survey question about how shelter management could support staff performing euthanasia. Recommendations included being supportive of staff, offering professional counselling, and rotating staff performing euthanasia. Through a survey to determine shelter managers’ perceptions of how their staff react to performing euthanasia, Anderson *et al.* (2013) reported that managers thought staff performing euthanasia were experiencing burn-out but did not believe this led to increased turnover.

The term ‘compassion fatigue’, a concept that was coined in the nursing literature to describe burn-out due to traumatic experiences (Joinson 1992), has been used to discuss what some shelter staff experience in their work, usually performing euthanasia. Schneider and Roberts (2016) conducted interviews about ‘occupational stress’, another conceptualised form of stress, with staff from seven USA and Canadian shelters and reported that in addition to euthanasia-related stress, staff discussed other stresses such as dealing with public perception of high euthanasia rates, negative encounters with human clients, and witnessing animal suffering, all of which contributed to what the researchers categorised as compassion fatigue. Levitt and Gezinski (2020) interviewed seven shelter staff about compassion fatigue and ‘resiliency.’ Andrukonis and Protopopova (2020) used the Impact of Event Scale-Revised to measure Post-Traumatic Stress Disorder and analysed occupational stress and ‘moral injury’ (an additional concept used to study trauma) using two scales (the Professional Quality of Life Scale and the Moral Injury Event Scale). They reported, for example, that ‘compassion satisfaction’ (i.e. pleasure from helping others through work) was positively associated with live-release rates (ie live animal outcomes divided by total outcomes). Finally, Figley and Roop (2006) focused on better ways to understand and assess compassion fatigue through surveys and Fournier and Mustful (2019) offered strategies for clinicians treating compassion fatigue.

A few researchers have written about how staff ‘cope’ with euthanasia-related stress. Frommer and Arluke (1999) observed shelter operations and conducted open-ended interviews with shelter staff and people relinquishing animals to the shelter. They framed their descriptive analysis on a theorised psychological defence mechanism known as ‘blame displacement’; the description concluded that relinquishers tended to blame others (e.g. family members, landlords) for having to relinquish their pet, or they blamed shelter staff if staff were unable to find a home for the animal, while shelter staff blamed relinquishers for being irresponsible or they convinced themselves that euthanasia may be better for animals than being abandoned. Through interviews with individuals working in animal sheltering or animal control, Reeve *et al.* (2004) used an event-based analysis to identify themes based on positive or negative ‘turning-point events’ that marked changes in how individuals felt about and coped with euthanasia-related work. Finally, Baran *et al.* (2009) used an open-ended survey to ask experienced shelter staff how they would advise less-experienced staff to cope with performing euthanasia. The main advice derived from the thematic content analysis was to express feelings, to avoid forming attachments to animals, and to acknowledge that in some cases euthanasia may be the best option.

In addition to euthanasia-related issues, there is a small body of research on the involvement of women in the sheltering and protection of animals, with a focus on how gender influences the work they do. For example, Markovits and Queen (2009) surveyed and interviewed dog rescue organisations in Michigan, USA, and concluded, based on statistical and discourse analysis, that women’s involvement in dog rescue was due in part to their sentimental, maternal and emotional nature. In a literature review focused on gender differences in human-animal interactions studies, Herzog (2007) noted the preponderance of women in animal protection work — a trend supported by demographic information in several studies (92% in Markovits & Queen 2009; 86% in Schabram & Maitlis 2017, and 100% in Schneider & Roberts 2016; see also Taylor 2010). In fact, Coulter and Fitzgerald (2019) described the “feminisation of animal control work”, arguing that this work has become gendered because the altruistic nature of the work draws many women.

Relatedly, Schabram and Maitlis (2017) applied a theory about work that may be termed a ‘calling’ — that is, work researchers characterise as a “meaningful beckoning toward activities that are morally, socially, and personally significant.” Applying this theory in an interview-based study, they identified different ‘calling paths’ that shelter staff took as they were doing and were challenged by their work (including, for example, euthanasia). Earlier, Taylor (2010) theorised that shelter staff do ‘emotion talk’ (i.e. expressing anger and compassion when speaking to staff about engaging with clients or caring for and euthanasing animals), in order to do ‘emotion work’ (i.e. managing their emotions). Although Taylor (2010) described the study as grounded in actual participants’ activities, the findings are abstracted into sociological theories about emotion management.

A few studies have addressed other aspects of shelter work. Surveys regarding animal intake procedures have studied disease awareness, screening and vaccination protocols (Steneroden *et al.* 2011; Spindel *et al.* 2013; Fagre *et al.* 2017) and the various interpretations of intake categories such as ‘stray’ (Vinic *et al.* 2019). Surveys have also been used to understand the relationship between shelter staff and veterinarians (Laderman-Jones *et al.* 2016) and to identify challenges of funding and facilities in dealing with horse abuse and investigations of neglect (Stull & Holcomb 2014). Arhant and Troxler (2014, 2017) used an approach-test (commonly used to test farm animals for their willingness to approach people) and a survey to assess the attitudes of shelter staff toward dogs and cats. Shelter staff had positive attitudes toward cats but there was no clear relationship between these attitudes and cat approach behaviour (Arhant & Troxler 2017). While staff generally had positive attitudes toward their work with dogs, the results of the approach-test proved difficult to interpret (Arhant & Troxler 2014).

### Focus group discussion about the literature on shelter staff and officers

In the opening ethnographic excerpt, a staff member sharing that they spend their breaks with fearful animals provides a glimpse into how shelter staff work with animals with behavioural problems to try and make them adoptable and avoid euthanasia. These activities did not deal with performing euthanasia, but rather the stress arising from euthanasia decisions and euthanasia prevention. Nonetheless, focus group participants were familiar with concepts from the literature, notably ‘caring-killing paradox’ and ‘compassion fatigue.’ This suggests that such conceptual understanding of the work is influential within the animal sheltering discourse.

In discussing these ideas, shelter staff and officers differed slightly from senior administrators. Shelter staff and officers talked about, and gave examples of, individual animals or people they encountered in their work that saddened or challenged them. In contrast, senior administrators talked about being unsure how to best support staff experiencing compassion fatigue caused by dealing repeatedly with people in difficult situations. They pointed out different programmes and counselling that exist, but they expressed concern that the staff and officers did not find the resources helpful.

Moreover, in contrast to how euthanasia-related stress is constructed in the literature, the participants emphasised stress arising from making decisions about euthanasia and their sense of helplessness when they could not rehabilitate or find a new home for an animal. In the organisation under study, where only veterinarians and veterinary technicians actually carry out euthanasia, the decision to euthanase (or save) an animal involves different work activities for individuals located differently within the institution. Shelter staff described their work as assembling information about animals (e.g. medical treatments and behaviour recorded on forms and checklists) for administrators and other people who make the decision. Senior administrators discussed the development and use of frameworks and protocols, such as the Asilomar Accords and Adoptability Guidelines (Gordon 2016) which provide detailed criteria for categorising animals based on their physical and behavioural health. This framework is meant to facilitate their decision, but administrators expressed that a lack of adherence to protocols generates tension between staff and/or between departments. The focus groups thus revealed that the *decision* to euthanase, which relied on co-ordinated work using institutional texts (a core interest of IE), was a more relevant topic for the participants than the actual euthanasia.

### Relinquishment of animals

*“When people call or email the shelter and need to relinquish their cat, we usually don’t have room and so we ask if they have a friend or family member that could take it or if they can wait a few weeks. If they can wait, then we send them the relinquishment forms so we can learn a little about the cat and we put them on the relinquishment list so we can call them when we have space.” [Comments by a shelter staff member detailing some of the initial work involved with ‘animal relinquishment’].*

As many animals are relinquished to shelters by their owners, the process of animal relinquishment is integral to the everyday work of shelter and animal protection staff. Typically, staff meet with people who bring an animal to the shelter, assess the situation and take people through the process of formally transferring ownership of the animal by signing a form. Relinquishment of animals has generated a large research literature; Coe *et al.* (2014) noted 192 citations on the topic, 44.3% of which were primary research articles published since 2006. Protopopova and Gunter (2017) reviewed research on interventions aimed at decreasing relinquishment of dogs by intervening either with the animals (e.g. training dogs to perform simple behaviours) or with adopters (e.g. providing training or educational materials). Researchers often call for a better understanding of relinquishment because many relinquished animals are euthanased (DiGiacomo *et al.* 1998; Salman *et al.* 1998; Weiss *et al.* 2014; Chua *et al.* 2017; Sandøe *et al.* 2019).

Most primary research articles on companion-animal relinquishment use surveys to collect data from people relinquishing animals. Several authors used data from the Regional Shelter Survey, an epidemiological survey carried out under the National Council on Pet Population Study and Policy (NCPPSP) in 1993 in the USA. Members of the NCPPSP and Salman *et al.* (1998) designed a standardised survey including a list of 66 potential reasons for relinquishing animals plus five additional reasons that the study participants gave during data collection. Subsequently, several studies built on the original survey to analyse and group common relinquishment reasons (Salman *et al.* 1998): relinquishment due to health and personal issues (Scarlett *et al.* 1999), moving (New *et al.* 1999), owner knowledge about and experience with animal behaviour (New *et al.* 2000), and relinquishment specifically for euthanasia (Kass *et al.* 2001). The literature focused almost entirely on the reasons for relinquishment, not on the work of staff who deal with relinquishers and relinquished animals.

In a somewhat different approach, DiGiacomo *et al.* (1998) used open-ended interviews to avoid what they termed the ‘one-word excuses of relinquishers.’ Moving and animal behaviour were common reasons for relinquishment. This research also reported that procrastination was a feature of relinquishers’ experience. Other researchers have developed original surveys that aim to understand relinquishment reasons in greater depth. For example, Weiss *et al.* (2014) used a 26-question survey and reported that most people in their sample cited a change in their household related to people or housing, not the animal’s behaviour, as influencing their decision to relinquish dogs. Using a survey based in part on previous research on relinquishment (e.g. Salman *et al.* 1998; Scarlett *et al.* 1999), Zito *et al.* (2016) reported that half of cat owners had multiple reasons for relinquishing their cat related to accommodation, personal factors and financial factors, while most non-owners (i.e. people relinquishing unowned cats) brought found cats to shelters, believing the cat would have better welfare in the shelter. Finally, other researchers have adapted and built upon questions from the 1993 NCPPSP survey and subsequent studies. Weng *et al.* (2006) adapted questions from New *et al.* (2000) to understand dog behaviour knowledge in a Taiwanese sample. They reported that more than 90% of participants thought that dogs misbehaved to ‘spite’ owners, higher than the 45% reported for the USA by New *et al.* (2000). Jacobetty *et al.* (2020) adapted scales (e.g. ‘general-trust-in-pets’ and ‘attitudes towards pet relinquishment’) and the relinquishment reasons reported in Salman *et al.* (1998) to construct a ‘motives-for-pet-relinquishment scale.’ ‘Pragmatic attitudes’ about relinquishment (i.e. rational, justifiable relinquishment) was correlated with past relinquishment (Jacobetty *et al.* 2020).

Some research on relinquishment has relied on shelter records where staff record the owner-reported ‘reason for relinquishment’ in databases that often allow only a single reason to be recorded. Cook and McCobb (2012) and Ellis *et al.* (2017) analysed USA and UK shelter records, respectively, and reported that common reasons for rabbit relinquishment included inability to provide care (or lack of time) and too many rabbits, with rabbit behaviour cited in some cases (Ellis *et al.* 2017). Casey *et al.* (2009) used open-ended responses from a standardised cat relinquishment form in the UK and grouped the responses into themes which included people ‘finding’ straying cats and ‘owner circumstances’ with sub-themes of ‘moving’, ‘owner death/illness’ and ‘financial problems.’ Alberthsen *et al.* (2016) reported that 91% of cat relinquishments in Australian Royal SPCA records were attributed to a category called ‘owner-related’ such as accommodation (i.e. pets not allowed) and having too many animals. Also in Australia, Hemy *et al.* (2017) reported that 29% of adult dog relinquishments were due to owner-related circumstances (e.g. moving, poor initial decision). Jensen *et al.* (2020) reported that reasons for relinquishment were most often ‘owner-related’ rather than ‘animal-related’ for both cats (75%) and dogs (74%), the most common reason being poor owner health. Similarly, using shelter records to generate themes, Shore *et al.* (2003) telephoned individuals who had relinquished an animal due to ‘moving.’ Most individuals (57 out of 67) confirmed they were moving, but also cited other factors such as landlord restrictions on pet ownership or pet size; seventeen respondents confirmed other relinquishment reasons that included ‘animal behaviour’ (Shore *et al.* 2003).

Researchers have also looked for relationships between animal intake data (including relinquishment) and census-based socioeconomic data. Rinzin *et al.* (2008) reported a positive but weak tendency for more cats and dogs to be brought to shelters from areas categorised as socioeconomically deprived by the New Zealand Deprivation Index. In Georgia, USA, Dyer and Milot (2019) reported that dogs from areas ranked high on the Social Vulnerability Index were at higher risk of being euthanased due to behaviours such as aggression and fearfulness which are often associated with social neglect. To investigate socioeconomic factors in geographic areas of high dog intake, Spencer *et al.* (2017) generated themes based on field observations and interviews with 39 community members and reported, for instance, that 40% of participants believed pet abandonment (which could lead to increased shelter intake) was due to: (i) inability to provide proper care for an animal; and (ii) uncontrolled breeding. Morris and Steffler (2011) compared relinquishment data and home-foreclosure data in California, USA, finding that while relinquishments and foreclosures were concentrated in the same areas, only one of the 248 relinquished dogs came from the address of a foreclosed house. Weng and Hart (2012), using relinquishment data from 2000 to 2010 in Chicago, determined that the economic recession of 2008–2010 led to an increase in the relinquishment of older dogs.

Costs of animal ownership and regulations that generate expenses have also been studied in relation to relinquishment of animals. Based on a survey, Dolan *et al.* (2015) concluded that animal cost, along with other factors, was strongly associated with the relinquishment of dogs. Similarly, Carter and Clark (2020) developed cost-related themes from interviews with people who had relinquished animals in Australia finding that relinquishment due to cost was mentioned, but only in combination with another factor, and that individuals often attempted to re-home their animal before taking it to the shelter. Others have performed statistical analyses on animal intake data over longer periods (eight to thirteen years) and concluded that a free (Kass *et al.* 2013) or subsidised (Scarlett & Johnston 2012) spay/neuter programme led to a decline in the number of cats brought into shelters. Sandøe *et al.* (2019) concluded that regulations that require dogs to be registered and controlled have decreased the number of dogs relinquished.

### Focus group discussion about the literature on relinquishment

In the opening ethnographic excerpt, the staff member’s description of what happens when a person calls to relinquish a pet provides a glimpse into practical aspects of this work. The work processes, and the tensions they may generate, were absent from the research literature on animal relinquishment which mostly aims to understand the reasons for relinquishment by surveying owners or analysing reasons recorded in shelter databases. For shelter staff, the relinquishment process typically begins with phone calls or emails from individuals who want to relinquish a pet. This requires shelter staff to listen, to evaluate the animal’s situation, to determine if the shelter has available space to house the animal, and to decide whether other resources (e.g. animal training, social service referral) might help. Such calls sometimes come from people who are struggling with complex social circumstances. Focus group participants described this work as challenging and sometimes (in the words of one participant) like managing a ‘crisis hotline.’ This detailed information about actual work processes is important in IE projects because it can help identify where tensions arise.

Animal protection officers’ work related to relinquishment typically involves visiting people’s homes in response to a report alleging abuse or neglect. Officers described how they follow set procedures to require compliance with applicable laws, and sometimes provide resources (bedding, food, referral to a free veterinary clinic) so that people can address problems. Their work is focused mostly on keeping animals and people together as long as animals are not in ‘distress’, but they may encourage relinquishment or remove animals where this is not achievable.

The work of both shelter staff and officers is guided by the need to maintain sufficient space in shelters as determined in part by the Capacity for Care (C4C) programme (Koret Shelter Medicine Program 2021). C4C calculates the optimal shelter animal population that can be provided with humane care based on available resources and other factors. This may require staff to triage cases and give priority to animals most at risk of harm such as animals rescued from abuse or neglect. Relinquishment by owners is the lowest triage level unless animals are at imminent risk of harm. Thus, according to the focus group participants, how the topic of relinquishment is handled in the research literature seems to have little relevance to the everyday ‘relinquishment work’ that shelter staff do.

Shelter staff also noted that the databases they use in their work have limitations when used for research. The database category ‘stray’, for instance, does not necessarily denote an unowned or unwanted animal. Staff are required to assign this category to animals that are abandoned outside the shelter, or that people bring to the shelter after finding them unattended. Staff also noted that there is no database category for the increasing number of animals relinquished by citizens who believe they have ‘rescued’ them from online sources (e.g. Craigslist, Kijiji) where animals are given away or sold.

### Length of stay in shelters

*“Henry’s been in shelter for 32 days now and I was wondering if we could put something on the website, generate pictures saying we have this kind of dog here available for adoption?”*

This quotation is from a daily staff meeting that included a brainstorming session on how staff might facilitate adoption of a dog named Henry whose length of stay (LOS, defined as the number of days between entering and leaving the shelter) had greatly exceeded the shelter’s average LOS of eleven days. Minimising LOS emerged as a key topic in the animal sheltering literature, and research has conceptualised the topic in two main ways. One acknowledges that many animals are euthanased if they are not adopted promptly and investigates LOS (or live-release rate) to reduce euthanasia by increasing adoptions (Brown *et al.* 2013; Protopopova *et al.* 2014; Gunter *et al.* 2016; Hawes *et al.* 2018; Patronek & Crowe 2018). The other, noting decreasing rates of euthanasia in shelters, is focused on reducing LOS because long periods in a shelter can contribute to physical and mental health problems for animals (Kay *et al.* 2018; Voslářová *et al.* 2019; review by Protopopova 2016). In either case, the goal of the research is generally to identify how shelters can adopt animals more quickly.

Many researchers have tried to identify adopter ‘preferences’ and other factors that may decrease LOS, often focusing on ‘phenotypic’ traits of animals such as breed group, sex, size, coat colour and age as listed in shelter databases. For example, Lepper *et al.* (2002), Brown *et al.* (2013), Brown and Morgan (2015), Kay *et al.* (2018) and Voslářová *et al.* (2019) used statistical analyses to compare animal traits to LOS, live-release rate (Patronek & Crowe 2018) or general outcomes of adopted, euthanased or transferred (Carini *et al.* 2020). A common finding from such analyses is that younger dogs have a shorter LOS than adults (Brown *et al.* 2013; Žák *et al.* 2015; Patronek & Crowe 2018). The evidence linking LOS to coat colour is more mixed. Two studies reported that black dogs had a longer LOS than white or yellow dogs (Kay *et al.* 2018; Voslářová *et al.* 2019), and one reported that white cats had a longer LOS than black cats (Miller *et al.* 2019). However, Patronek and Crowe (2018) reported no difference in live-release rate for different coloured dogs, and both Sinski *et al.* (2016) and Carini *et al.* (2020) concluded that coat colour was not a significant predictor of outcome (adoption, euthanasia, or transfer) for either dogs or cats. Studies often conclude that understanding shelter ‘context’ and adopter preference is important (Brown *et al.* 2013; Carini *et al.* 2020).

Some authors have related LOS to other information in shelter databases, often using statistical analysis to interpret findings. Noting the source of animals, Notaro (2004) reported that animals brought to shelters by animal control officers had a longer LOS than animals brought by the public. Hawes *et al.* (2018) used categorical information from animal intake (e.g. body condition, health problems) to determine what influenced LOS for older animals. Patronek and Crowe (2018) reported that dogs in foster homes or dogs returned after an unsuccessful adoption had increased odds of live release while Kay *et al.* (2018) determined that dogs in shelters located in larger human population centres had faster adoption times than those in smaller centres.

Other approaches have also been used to study LOS. Protopopova *et al.* (2014) video-recorded dogs and analysed the types of behaviour in the kennel that influenced LOS; they concluded that LOS was higher for dogs leaning on the wall, facing away from the front of the kennel and standing. Other authors have investigated how LOS is influenced by aspects of shelter organisation. Using statistical analysis of Canadian shelter data, Janke *et al.* (2017) and Karsten *et al.* (2017) concluded that implementation of the C4C programme decreased LOS for cats. A case study report about C4C implementation in Canada provided additional insights by asking and reporting what shelter staff considered worked well (e.g. additional cage space for cats) and did not work well (e.g. visitors being bitten when staff were not present in cat adoption rooms) (Humane Canada 2018). Weiss *et al.* (2013) reported improved live-release rates for cats and dogs in ASPCA shelters that had ‘partnerships’ with other animal protection and rescue agencies, although they did not include details about the actual work processes and activities of the partnership that might explain the improvement. In addition, Abrams *et al.* (2020) reported that dogs in a New York shelter were more likely to be adopted if they received the antidepressant trazadone.

Finally, a few researchers have investigated how potential adopters ‘perceive’ animal photographs, profiles and breed labels. Using data available through existing online pet-adoption platforms some authors have explored how dog photographs (Lampe & Witte 2015; Nakamura *et al.* 2020) or written profiles (Nakamura *et al.* 2019) affected speed of adoption. Rix *et al.* (2021) concluded that cat profiles written in the third person (rather than the first person) were associated with shorter LOS, while Nakamura *et al.* (2019) focused on specific words used in dog profiles. For example, Staffordshire Terriers and Jack Russell Terriers had the shortest LOS when the word ‘gentle’ was used. Interestingly, Lampe and Witte (2015) reported that dogs photographed outdoors were adopted more quickly while Nakamura *et al.* (2020) reported that dogs photographed in a kennel setting were adopted more quickly. Gunter *et al.* (2016) designed a study that used shelter database records and an experiment that showed members of the public photographs of pit-bull-type dogs and ‘lookalike’ dogs with and without labels to identify the breed. They reported that a label of ‘pit-bull’ could increase LOS because of a negative perception of the breed. Finally, Cohen *et al.* (2020) examined shelter records before and after the removal of breed labels and reported that removal of breed labels for dogs decreased LOS.

### Focus group discussion about the literature on length of stay

In the opening ethnographic excerpt, the staff member proposing how to facilitate the adoption of a dog provides a glimpse of their concern over an individual animal with an extended LOS in the shelter. Noting that much of the research concentrates on animal characteristics that influence LOS, focus group participants identified other features of their work that affect LOS but are not covered in the literature. This work, which was completed mostly by shelter staff, included what they described as ‘veterinary treatment’ and ‘behavioural modification’ of animals.

Regarding veterinary treatment work, both shelter staff and senior administrators noted that the need to follow protocols for veterinary interventions (e.g. treating ringworm) or referring animals to specialists took time and thus increased LOS for animals. Regarding behavioural modification work, senior administrators noted that animals currently being relinquished seem to have more behavioural problems than in years past yet there are not enough trained staff to carry out behavioural modification. Shelter staff, however, reported feeling responsible and sometimes compelled to do behavioural modification work so animals could be adopted more quickly, for instance by sitting with fearful animals, bringing food rewards so the animals would associate people with positive occurrences, and habituating dogs to wearing collars and leashes. Officers also recognised that staff had limited time in their daily work to do what they knew about and talked about as behavioural modification. This influenced their efforts to try and keep animals with their owners, when possible, for example by donating supplies. These instances of feeling pressure, responsibility and a lack of time are tensions that could be explored through IE.

Finally, shelter staff and officers identified a need for research on how transfer programmes affect animal behaviour and health. Transfer programmes aim to decrease LOS by moving animals to larger centres with more adopters, but also involve additional work activities such as preparing kennels, performing medical intake procedures and moving animals around within the shelter to accommodate those with special needs.

## Discussion

In this review, we attempted to identify how published research approaches and ways of thinking seen in the research literature have shaped the current understanding of animal sheltering, and how the research literature relates (or not) to problems and challenges identified by people doing the actual work of animal sheltering.

The growing literature on euthanasia-related stress, which has received much attention since the 1990s, shows that this topic remains a strong focus of research framed by concepts like compassion fatigue and the caring-killing paradox. Scientists are now using established social-science research methods, such as standardised questionnaires and psychometric constructs, to build on prior knowledge, with the proposed aim of understanding the phenomenon as it is currently conceptualised. When Arluke (1991) and Arluke and Sanders (1996) began to report study findings, euthanasia of shelter animals was very common in North America, but it is now declining sharply. In Canada, for example, the percentage of animals euthanased in shelters declined between 1993 and 2019 from 30 to 10 percent of dogs and from 60 to 15 percent of cats (Humane Canada 2021). In the USA, a survey in 1973 reported that 13.5 million animals were euthanased (Rowan & Kartal 2018) and estimates remained high in the late 1980s and early 1990s (Bartlett *et al.* 2005); a more recent estimate, however, reported that 920,000 cats and dogs are currently euthanased each year (ASPCA 2021a). The continued strong focus on euthanasia-related stress may be an example of how, once a topic has become established in the research literature, it can continue to be a conceptual focus of study despite what is actually happening in sheltering work.

Focus group participants were fluent in the language of euthanasia-related stress and compassion fatigue and they used these terms in relating their own experiences. Indeed, institutional ethnographers look for traces of how topics within the academic discourse circulate in institutions, discovering how people actively participate in them through their use of texts and language in their everyday work (Smith 2005; p 224). However, the participants — working in an institution where the modest number of euthanasias are done by veterinary staff — noted that the challenges are now very different from how they are represented in the research literature. For focus group participants, the stress associated with euthanasia was linked more with making, discussing and sometimes defending the decision to euthanase rather than conducting euthanasia routinely. To better understand these tensions, more attention is needed on the actual work involved in an animal’s institutional pathway that results in euthanasia.

In the research literature, descriptions about the work involved with performing euthanasia are broad. Arluke (1991) recognised two distinct roles: the ‘holder’ who controls the animal and the ‘shooter’ who administers the injection. Baran *et al.* (2012) also positioned euthanasia as a work process but also discussed the work as categorical ‘roles’ that included selecting animals for euthanasia and confirming death. Both these authors suggested that these specific work procedures may lead to different responses and levels of stress. In this research, however, euthanasia is categorised as a distinct ‘episode’ of work — disconnected from the series of events and decisions that contribute to a final decision to euthanase. Moreover, most studies have identified shelter staff simply as those who ‘participate in’ (White & Shawhan 1996), are ‘involved with’ (Reeve *et al.* 2004, 2005), ‘perform’ (Rogelberg *et al.* 2007a; Anderson *et al.* 2013), are ‘directly involved with’ (Baran *et al.* 2009) or have ‘direct or indirect contact with’ (Lopina *et al.* 2012) euthanasia. Attention to the actual work activities that lead to a decision to euthanase (or not), rather than the use of generic categories, could lead to greater insight into how tensions and conflict may arise.

While most existing research on compassion fatigue in shelters is focused on euthanasia, focus group participants noted the emotional toll on staff and officers whose work involves dealing with people in ‘distress.’ This work often includes listening to people and referring them to available services — tasks that are more related to social work than to traditional animal sheltering yet are important for the widely accepted priority of keeping people and animals together (e.g. LaVallee *et al.* 2017; Baker *et al.* 2018). Some research has discussed shelter staff interacting with relinquishers (DiGiacomo *et al.* 1998; Frommer & Arluke 1999; Irvine 2003) and with the public (Schneider & Roberts 2016) but the focus has been on negative interactions. An IE approach would shift the focus to examine the actual work staff and officers do to keep people and their animals together including co-ordinating and collaborating with social service agencies that may also be involved.

Only a few research studies on shelter staff have applied the methods of ethnography rather than relying on questionnaires and scales. Taylor (2010) used observations and interviews with shelter staff to understand how they expressed emotions. However, unlike an IE approach that stays firmly connected to how happenings and experiences are organised, findings are analysed within sociological theories about ‘emotion management.’ Irvine (2003) made ethnographic observations of the relinquishment process and conducted in-depth interviews with people relinquishing animals. She pointed out that the current understanding of relinquishment may be limited by research relying on information from standardised intake forms or a drop-down list of relinquishment reasons in a database (following, for example, Salman *et al.* 1998; New *et al.* 1999; Kass *et al.* 2001) or if the records allow people to report only a single reason for relinquishment. Irvine’s misgivings over the current knowledge about relinquishment is supported by a review by Protopopova and Gunter (2017) who noted that much of the understanding about dog relinquishment has been influenced by the 1993 NCPPSP survey. As noted by Levitt and Gezinski (2020), future research should expand to include other aspects of animal shelter work beyond euthanasia “to gain a more holistic understanding of [workers’] experiences and needs.”

In discussing relinquishment, the focus group participants suggested that understanding may be limited by the prevailing reliance on shelter data which often were not collected with research purposes in mind. For example, the term ‘stray’ in shelter data is sometimes used for various categories of animals (Zito *et al.* 2016; Vinic *et al.* 2019). Shelter staff reported that owners often express multiple reasons for relinquishing an animal, and it is difficult to select a single reason as required by the database. ‘Following up with relinquishers’, as done by Shore *et al.* (2003) and Irvine (2003), could also help overcome the limitations inherent in the use of shelter records for research.

Like research on relinquishment, conventional statistical research on LOS uses shelter database records and this approach has organised how LOS is understood. Shelter records are typically collected to track average LOS for the purpose of generating annual reports and information for the organisation. However, certain variables (e.g. breed, coat colour) may be recorded in an inconsistent way (Kay *et al.* 2018; Patronek & Crowe 2018; Carini *et al.* 2020), a point also discussed by shelter staff in the focus groups. Thus, the variables recorded, and hence used by researchers, may be entered inconsistently and may not be the most important determinants of LOS. Detailed descriptions of the broad work processes being undertaken in shelters (that lead to a lengthy or reduced LOS) are overlooked in the current published approaches.

Focus group participants clearly appreciated LOS research that could help shelters achieve more prompt adoption. However, their own knowledge about the protocols related to animal health (e.g. treating fungal infections, collecting samples for testing) which increase LOS is excluded from current published work. The large ‘shelter medicine’ literature on prevention and treatment of disease (excluded from this review) mainly reports animal-based outcomes such as upper respiratory infection (e.g. Gourkow *et al.* 2013) and enteric parasites (e.g. Villeneuve *et al.* 2015). An alternative approach would be to shift attention to descriptions of the everyday work routines and knowledge of staff involved with animal health and care to more effectively support their work.

Research on LOS has tended to emphasise adopter preferences and effective ways to present animals to potential adopters. Focus group participants, however, emphasised the increasing number of animals with behavioural problems that prevent prompt adoption and require the extra work of behaviour modification. Behaviours that need to be modified are identified in part through formal behavioural ‘assessments’, but the validity and utility of such assessments is debated (Patronek & Bradley 2016). Importantly, the actual work involved in performing such assessments is less clear although Mornement *et al.* (2010) noted through interviews that shelter staff thought the assessment could be improved by assessing more behaviours and having more time for the assessment.

The work of conducting behavioural modification in shelters has been explored by some researchers (e.g. Orihel *et al.* 2005 and Orihel & Fraser 2008 on inter-dog aggression; Mohan-Gibbons *et al.* 2012 on food guarding; Marder 2013 on behavioural pharmacology), and animal sheltering organisations have created training materials on this topic (e.g. ASPCA 2021b). Recent research has reported that fearful animals from hoarding situations require more time from shelter staff (McMillan *et al.* 2016; Strong *et al.* 2019). A more nuanced understanding of the actual work involved with behavioural modification (e.g. how behavioural modification is done, by whom, the training required) and a better understanding of the benefits that are accrued by informal human-animal interactions could give insight into how shelter staff monitor the progress animals make toward becoming adoption candidates and how that daily work contributes to the ‘adoptability’ decisions about animals in care.

## Animal welfare implications and conclusion

The research literature on animal sheltering, while involving many fields such as veterinary medicine and social sciences, tends to be organised around a few key theories and frameworks. Many studies apply high-level concepts (compassion fatigue, euthanasia-related stress) and attempt to quantify these through standardised scales. Studies on animal relinquishment often use standardised questionnaires or shelter database records to understand the reasons why people relinquish animals. Studies on LOS commonly rely on shelter database records to understand animal characteristics that increase time spent in the shelter.

An institutional ethnographic approach would complement such research by shifting the focus to observing the actual work practices of shelter staff and officers and identifying how texts are taken up in their work. It would focus on what is difficult and challenging in these work practices — with both people and animals — and then track the social organisation of those tensions. It would map and track the actual work involved with making decisions to euthanase and identify how those decisions create tensions for staff. It would describe the actual practices involved in the relinquishment of animals and the processes that owners, staff and animals are subject to. From here, policies, protocols and routines could be modified to better serve the interests of the animals and the staff. Finally, it would complement metrics on LOS by identifying the work practices that lead to shorter or longer LOS for individual animals whose stay far exceeds the average.
